# Self reported health status, and health service contact, of illicit drug users aged 50 and over: a qualitative interview study in Merseyside, United Kingdom

**DOI:** 10.1186/1471-2318-9-45

**Published:** 2009-10-09

**Authors:** Caryl M Beynon, Brenda Roe, Paul Duffy, Lucy Pickering

**Affiliations:** 1 Centre for Public Health, Research Directorate, Faculty of Health and Applied Social Sciences, Liverpool John Moores University, Castle House, North Street, Liverpool, L3 2AY, UK; 2 Evidence Based Practice Research Centre, Faculty of Health, Edge Hill University, St Helens Road, Ormskirk, Lancashire, L39 4QP, UK; 3 School of Health and Social Care, Oxford Brookes University, Jack Straw's Lane, Oxford, OX3 0FL, UK

## Abstract

**Background:**

The populations of industrialised countries are ageing; as this occurs, those who continue to use alcohol and illicit drugs age also. While alcohol use among older people is well documented, use of illicit drugs continues to be perceived as behaviour of young people and is a neglected area of research. This is the first published qualitative research on the experiences of older drug users in the United Kingdom.

**Methods:**

Semi-structured interviews were conducted in Merseyside, in 2008, with drug users aged 50 and over recruited through drug treatment services. Interviews were recorded and transcribed and analysed thematically. Only health status and health service contact are reported here.

**Results:**

Nine men and one woman were interviewed (age range: 54 to 61 years); all but one had been using drugs continuously or intermittently for at least 30 years. Interviewees exhibited high levels of physical and mental morbidity; hepatitis C was particularly prevalent. Injecting-related damage to arm veins resulted in interviewees switching to riskier injecting practices. Poor mental health was evident and interviewees described their lives as depressing. The death of drug-using friends was a common theme and social isolation was apparent. Interviewees also described a deterioration of memory. Generic healthcare was not always perceived as optimal, while issues relating to drug specific services were similar to those arising among younger cohorts of drug users, for example, complaints about inadequate doses of prescribed medication.

**Conclusion:**

The concurrent effects of drug use and ageing are not well understood but are thought to exacerbate, or accelerate the onset of, medical conditions which are more prevalent in older age. Here, interviewees had poor physical and mental health but low expectations of health services. Older drug users who are not in contact with services are likely to have greater unmet needs. The number of drug users aged 50 and over is increasing in Europe and America; this group represent a vulnerable, and in Europe, a largely hidden population. Further work to evaluate the impact of this change in demography is urgently needed.

## Background

The world's population is ageing. In 1900, the global population was estimated to have only 1% of people aged 65 years and over, by 2000 this figure was 7%, and by 2050, the estimated proportion will be 20% [1]. As our populations age, people who continue to use drugs and alcohol age also. Projections from the United States of America (USA), for example, suggest that the number of people aged 50 and over who need treatment for drug and alcohol problems will increase from 1.7 million in 2000 to 4.4 million in 2020 [2], while estimates from Europe suggest that the number of people aged 65 and over needing treatment for such problems will more than double between 2001 and 2020 [3]. In the United Kingdom (UK), research using established monitoring systems has shown that the proportion of drug users aged 50 and over in contact with specialist drug services in Cheshire and Merseyside (the only area of the country to have collected data on drug treatment clients consistently since 1998 and therefore the only area able to monitor demographic changes) has increased between 1998 and 2004/05 from 1.5% to 3.6% (P < 0.001) and 1.9% to 3.2% (P < 0.001) for men and women respectively. Furthermore, the proportion aged 40 to 49, potential older drug users of the future, increased from 8.1% in 1998 to 19.6% in 2004/05 [4].

A prospective cohort study undertaken in the USA demonstrated that older drug users have levels of morbidity considerably higher than the general population [5], while qualitative research also completed in the USA demonstrated that they experience loneliness, stress and fear of victimisation [6]. The use of illicit drugs is, however, still largely perceived to be behaviour of the young and, consequently, continues to be a neglected area of research [7]. In particular, there has to date, and to the best of our knowledge, been no qualitative research published on the experiences of older drug users in the UK. Information relating to what drugs older people in the UK use, the reasons for initiating or continuing drug use and the specific health and treatment needs of this group of people is, at present, lacking in the UK; our understanding can not be conceptualised using data from the USA due to cultural, legal and attitudinal differences between the two countries, and differences in the manner in which healthcare is delivered [8].

The aim of this present study was to undertake an investigation into issues salient to older drug users. More specifically, the study aimed to identify substance (drug and alcohol) use, self-reported health status and contact with generic and specialist health services of a cohort of older drug users in contact with specialist drug treatment services. Here we define older drug users as those aged 50 and over.

## Methods

Qualitative research was undertaken using semi-structured interviews with prompts to capture older people's experiences across the life course. These interviews covered a range of topics but only results pertaining to health status and health service contact, plus brief contextual data, are presented here. While the interviewer had a number of pre-determined topics to cover, the interviewer was sensitive to the interviewee and their narrative, allowing for the exploration of topics that arose spontaneously, in order to enhance the qualitative information obtained. Interviews were conducted by the same researcher (LP) to ensure consistency and all were taped and transcribed. LP also provided additional information to enhance the quality of the transcriptions. Approval for this study was granted by the Liverpool John Moores University Ethical Committee. Each participant received a £10 shopping voucher in compensation for their time dedicated to the study.

### Recruitment

Staff working in voluntary sector, specialist drug treatment services in Merseyside were asked to identify clients within their care aged 50 and over and to introduce the study to them. People who expressed a willingness to participate were given an information sheet to take with them and the member of staff contacted the research team with the client's name and contact telephone number. A member of the research team (LP) made contact with the potential participant and organised a convenient time and place for the interview to occur. Prior to the interview, each potential participant was given another information sheet, and a consent form. Full informed consent was obtained, including specific consent to use anonymised quotes, and it was explained that participation would not influence their ongoing medical care. Interviews were conducted between 16^th ^January and 22^nd ^February 2008. Recruitment continued until no new major themes were identified.

### Analysis

Transcripts were scrutinised by all four authors, who analysed them using thematic analysis [9]. Each member of the research team initially independently identified broad themes which were given codes; these were then discussed and agreed by the research team. Each transcript was then read again by all authors, were coded, and passages relating to each theme were grouped together. Themes included drug use, health, quality of life, lifestyle and employment, relationships and social networks and use of services.

### Reliability and validity

The first three interviews were used to establish the feasibility of recruitment, inclusion criteria and to test the methods of data collection. No changes to the interview schedule or prompts were made but we reduced the minimum age from 60 to 50 years because it proved difficult to recruit a sufficient number of people aged over 60. Thematic analysis showed no difference in the themes and sub-themes between these pilot interviews and the data from all other interviews and so the pilot interviews have been included in the results. Where interviewees provided contradictory or revisionist statements, the interviewer (LP) sought clarification in order to enhance the validity, accuracy and consistency of responses and our understanding of their discourse. Reliability of the analysis was assured by members of the team reading and re-reading transcripts, and by discussion and agreement of the themes and sub-themes. The measures described here are standard verification techniques for establishing quality, validity and rigour in qualitative research [10,11].

## Results and Discussion

### Context, demography and substance use

Nine men and one woman were interviewed in a range of settings; within a doctor's surgery (n = 2), at home (n = 2), in a care home (n = 1) and at a drug treatment service (n = 5). The ages of interviewees ranged from 54 to 61 years. Six were single and four were divorced at the time of the interview, with two living with a male friend who acted as a carer, one more who lived next door to a friend/male carer and another who shared their accommodation with other drug users. Their accommodation varied; some lived in a hostel, others in their own home (council house, flat or housing association bed sit), one man lived in a care home and another lived in a caravan.

All but one interviewee had started substance use when in their adolescent, or early adult, years but there was no single pattern to drug initiation; drugs first used included alcohol, cannabis, lysergic acid (LSD), amphetamine, morphine hydrochloride, heroin, 'speedballs' (heroin in combination with a strong stimulant, usually cocaine) and (psilocybin-containing) mushrooms and people simply switched drugs according to availability. For these people, drug use across the life course was divided into three camps, with some interviewees ceasing drug use for many years at a time, some using drugs intermittently with short periods of abstinence, while others followed a course of near continual consumption. Conversely, one interviewee was a late onset user, having commenced drinking aged 30 (which progressed into problematic drinking) and drug use (heroin) aged 46. Every interviewee was, or had been, a problematic drug user (currently defined in the UK as a user of opiates and/or crack cocaine [12]). Every interviewee had used heroin during their lives and some were still using this drug at the time of interview; many were in receipt of a methadone prescription as part of opiate substitution therapy. Six people also reported a history of cocaine and/or crack cocaine use. Some interviewees acknowledged that they had injected drugs or inferred that this was the case by discussing injection-related health consequences. A more detailed discussion of substance use and other themes arising from this study will be presented elsewhere.

### Physical health

The effects of the interaction between drug use and those processes that characterise ageing are poorly understood. However, there is evidence to show that drug use exacerbates, or accelerates the onset of, medical conditions which are more prevalent in older age. Stimulant use, for example, can lead to cardiovascular complications in younger drug users, while longer term use may increase the likelihood of premature atherosclerosis, ventricular hypertrophy and cardiomyopathy. Drug use has also been associated with an earlier onset on diabetes, while neurological disorders, respiratory disorders, cancer and other age associated diseases may also all be worsened by drug use [13]. Furthermore, the disorientating or hallucinating effects of some psychoactive drugs increase the risk of accidents and falls, particularly in combination with prescribed or over-the-counter medications [13,14]. Falls are a major cause of disability and mortality in older people [15] and can be presumed to occur even more frequently among those who use psychoactive drugs including alcohol.

Our interviewees reported a high level of physical morbidity; circulatory problems, respiratory problems, pneumonia, diabetes, hepatitis and liver cirrhosis. Overdose and injuries also featured in their narratives. Summarising, one man stated; *"I'm... handicapped to the extent of can't go out, suffer with arthritis in the right leg and lower back, blind in one eye, suffer hepatitis C...."*. Hepatitis C infection, contracted by injecting drug users through sharing contaminated drug injecting and/or preparation equipment [16], was a common theme, with half of those interviewed stating they were infected with the virus. Despite being a serious infection, progressing to cirrhosis, end stage liver disease and, in about one to five percent of those chronically infected, hepatocellular carcinoma [17], a synthesis of qualitative research demonstrated that drug users of all ages tend to see hepatitis C as somewhat of an inevitability of injecting, that being infected is normal and that it is physically benign, particularly relative to HIV [18]. This perception of its benignity is presumably even greater among those older drug users who contracted this infection many years previously and have experienced no adverse consequences of long term infection. Certainly our interviewees did not perceive their infection to be problematic;

*"I've never really had any serious illnesses.... I forgot the hep C y'know. I've had no symptoms. I haven't been sick or anything y'know, but I've got the virus"*.

There are many factors which influence the aetiology of hepatitis C infection and its impact on liver function and drug users of all ages are infected with the virus in the UK. However, those older drug users who contracted the virus many years previously may be at an advanced stage of disease progression. Furthermore, younger age at the commencement of treatment is one positive predictor of success in clearing the virus [19] and healthcare services will need to be prepared to care for older drug users who may be disproportionately affected by treatment failure. Treatment services need also be aware of the wider determinants impacting on the likelihood of older drug users adhering to treatment and in particular the role of social support in coping with the relatively long and unpleasant nature of hepatitis C treatment [20]. Qualitative research from the USA reported that older drug users had severed links with non-drug using family and friends over the years of drug taking [6], a premise supported by the current research. For older drug users with little or no family support, the involvement of services to support a person while undergoing treatment will play an important role [20].

The high proportion of interviewees with hepatitis C reflects findings from the USA, where medical examinations of 108 older heroin-dependent men (mean age 58.4 years) showed that 95 percent of their subjects had been exposed to the hepatitis C virus, while more than half were found to have abnormal liver function; these rates were much higher than found in the general population [5]. In recent years, the enormity of the UK's hepatitis C epidemic has been evidenced through a number of sources, with the Health Protection Agency consistently citing it as the most significant infectious disease affecting people who inject drugs [19]. There are currently an estimated 330,000 problematic drug users (users of opiates and/or crack cocaine) in England and 120,000 current injectors of these substances [21] plus many others who are ex-drug injectors. According to Health Protection Agency estimates, approximately half of all current drug injectors are infected with hepatitis C, and both incidence and prevalence continue to rise [19]. As the already ageing cohort of drug users [4] age further, those infected with the virus will gradually develop symptoms of the infection and the full impact of hepatitis C infection among current and ex- drug injectors will become apparent. Currently, the burden of drug related chronic conditions such as hepatitis C, which disproportionately affect older drug users compared to their younger counterparts, is not being fully recognised because such deaths are not classified under UK or European definitions as drug related deaths and are therefore not counted in official figures [22,23].

Four interviewees discussed vein damage as a consequence of long term intravenous drug use and the fact that this made continuing to inject in their arms difficult. Consequently, interviewees had switched to more risky injecting practices (which included injecting into their groin and feet [24]) to overcome problems of gaining venous access, despite being aware of the elevated risk such practices convey;

*"It's harder now to inject because your veins. Your veins sort of go y'know... I've been injecting in my foot, which is stupid really, y'know. You can catch an infection and you could end up losing your leg"*.

One man talked of having been hospitalised for deep vein thrombosis (a blood clot which forms in a deep vein), a recognised potential consequence of intravenous drug use for all ages [24]. However, deep vein thrombosis becomes more likely among older injectors, particularly for those who have been heavy smokers, because in addition to the damage to veins caused by injecting, the ageing process is associated with changes in blood pressure, venous valve deterioration and reduced regenerative process [25]; healthcare staff should ensure that older drug users, especially those who smoke, are aware of this increased risk.

Only three of the 10 interviewees complained of having respiratory problems despite research from the USA showing heroin-dependent males to have high rates of obstructive lung disease [5]. This difference may be due to the men recruited in the USA study being predominantly smokers of heroin while the majority of those interviewed in this study discussed injecting as their mode of drug administration. One man interviewed in the present study was a heavy crack cocaine smoker and complained of respiratory problems and how this affected him; *"I get out of breath easy. I absolutely panic then"*. A second crack cocaine smoker, who had experienced bronchial pneumonia twice, concurred; *"it's tender inside and when I breathe I can't breathe hard cos it hurts like hell"*. The link between smoking any substance, including cigarettes, and respiratory complications is well known. Clinical guidelines recognise the high prevalence of smoking-related disorders among drug users, and the importance of offering smoking cessation interventions for smokers [26].

The people interviewed in the present study had used a variety of drugs across their life course, including alcohol, and had complex physical health needs. Drug use into older age can be seen as accelerating the physical health changes commonly associated with ageing. Furthermore, immunosenescence, the notion that there is an age related dysfunction of the immune system which leads to enhanced risk of infection [27], is an issue for all older people but will presumably have greater consequences for those older drug users who have experienced a lifetime of deprivation and poor environmental condition, making them particularly susceptible to opportunistic diseases such as pneumonia and other infections when compared to younger drug users or older people who do not use drugs [22]. Moreover, chronic poor nutrition will compound the physical problems arising from concomitant drug use and ageing; *"I don't know but I think I'm seven stone now... I only have chips seven days a week"*. Consequently, for older drug users who have survived overdoses and other negative acute effects of drug use, it appears to be the longer term effects of drug use that become important in terms of their impact on physical health. Healthcare services, including drug specific services therefore need to recognise the impact of chronic multiple drug use on the physical health of older drug users and offer a package of care that addresses the person's general health in addition to the provision of drug treatment [22].

### Mental health

The links between mental health and substance use are complex but well documented and a high prevalence of co-morbidity exists. One study conducted in the UK, for example, reported that 44 percent of patients in community mental health services reported problem drug use and/or harmful drinking in the previous year, while 75 percent of people in contact with substance misuse services reported a psychiatric disorder during the same period of time [28]. Psychological conditions, including depression, loneliness, anxiety, memory problems, cognitive impairment, dementia and confusion become more prevalent in older age [15]. Ageing may therefore be associated with further elevation in the prevalence of mental health disorders in a population where the prevalence of such conditions is already considerably higher than that of the general population. Furthermore, prolonged use of some psychotropic drugs for example the benzodiazepines, is actually associated with depression and cognitive decline [13]. Under-detection of such conditions including depression is relatively common among all older people [15] but may be a more significant issue for drug users because a considerable proportion do not seek treatment in services where screening for such disorders occurs [29].

Four interviewees commented that, during the course of their lives, their drug use had started, escalated or resumed following significant stressful events. For example, when discussing a relationship break up one man said; *"You reach a point in our life, different points in your life where you've got a lot of stress and the easiest thing in the world is to say 'oh fuck it'. And once you've said that, if you're a drug user, a drug abuser, once you've said that, you're on the rocky road to destruction again"*. Across the life course, feeling sad or depressed due to stressful life events accounted for the reason why some of our interviewees recommenced drug use following long periods of abstinence and that using drugs was a means to forget about worries; *That's all we do anyway, just take it *[heroin] *and just mong *[be relaxed and docile] *for the day like and forget about your worries and everything, got problems and you forget all that. Things like that; it's the reason why people take it like"*.

These findings highlight the need for healthcare services to be vigilant for drug and alcohol use by older adults, even among those who have ceased use for many years, while social support services for older people should also be aware of the need to screen people for drug and alcohol use, particularly those in contact during times of stress such as occurs following bereavement or relationship failure. However evidence from the USA suggests that healthcare professionals are currently poor at diagnosing substance use among older people [30] possibly because of a lack of awareness that substance use among older adults occurs. Furthermore, the current screening tools for drug use, for example the Diagnostic and Statistical Manual of Mental Disorders IV for substance abuse, have not been validated for older populations [13], furthering problems of diagnosis.

Interviewees talked in terms of a life using drugs being depressing; *"It's a depressing life y'know. It's no good" *and how life not using drugs (where this was the case) had been more positive; *"I was high on myself y'know *[when not using drugs]. *I'd look in the mirror and see somebody I loved. I don't any longer.... I can't see a road back to loving myself again"*. Conversely however, two interviewees were comparatively content with life at the time of the interview, with one recognising a link between reduced drug use and improvements in mood; *"The ale was just a depressant wasn't it? And the heroin was just a depressant. Just making myself lower and lower all the time, wasn't I? Now I'm off the two of them I'm happy as a lark"*.

The loss of friends, predominantly through drug use, was a common theme through the interviews and was also associated with feelings of sadness and depression; this finding is unsurprising in light of extensive evidence to show that strong social networks have a positive effect on the health of older people, particularly on their mental health [31]. Loss of friends, isolation and changes in social networks are issues experienced by many older people [32] but this is intensified among drug users and occurs earlier in life because rates of mortality are higher than in the general population [33]. Furthermore, older drug users tend to associate exclusively with other drug users, having severed links with non-drug using family and friends over the years of drug taking [6], a premise supported by the findings of our interviews. Consequently, survivors are left alone or in contact with younger drug users from a different peer group. One particularly erudite man who had recently experienced the last of his friends die said;

*"It's made me extremely depressed in as much as the few that were left are people I could talk to and at least there's consolation in company if its good company..*. [now] *the only company I could find if I wanted to would be people who are younger and on that totally different scene, and like I say, I have nothing in common with them... Consequently find myself quite lonely at times"*.

These results confirm findings from the USA, which highlighted isolation and loneliness as important issues for older drug users [6]. For one interviewee, isolation was heightened by being morbidly obese and unable to walk more than a few steps; a physical state derived from cocaine-induced agoraphobia and inactivity;

*"But I got so heavy with it *[cocaine use] *that I ended up getting agoraphobia cos I spent, must have been nearly four years in bed, just in bed...I think it was the four years what I ended up spending in bed that sort of got me into the state of putting on so much weight"*.

Furthermore, the isolation experienced by some older people may be exacerbated among older drug users by drug-related paranoia, experienced by three interviewees; *"I don't like going to pubs. I always get paranoid... Everybody's looking at me... Drugs do it to you"*.

Younger drug users also experience friends dying (particularly through acute drug toxicity) but they may be somewhat cushioned from the effects by having larger social networks and more opportunities to make new friends. Younger drug users are also more likely to be in contact with their families. As older drug users die, the pool of peers grows ever smaller for those older drug users left behind. Of interest to this discussion, three interviewees appeared to have overcome this problem by forming new support structures, through developing friendships with much younger drug users who acted as carers. As the mobility, physical and mental capacity of older people fade they need to develop contacts with younger people to secure emotional and practical support. This is a considerable challenge for those without children [31], yet it appears that symbiotic alliances between older drug users and younger drug users develop out of this practical necessity. For example, one interviewee who had no family, had seen most of his friends die, who had recently had a stroke and who rarely left his house relied heavily on a much younger friend for assistance with daily tasks like collecting shopping, his benefits and his methadone prescription and doing the cooking. His younger friend, who was present during the interview comments *"he helps me as I help him. We're good for each other"*. The younger drug user was not the focus of the interview and how exactly he benefited from this relationship was not explored although he did say that he enjoyed the company of the older drug user and suggested that he used his older friend's home as a preferable place to use drugs despite having his own place to live; *"I'd rather do it *[use drugs] *here *[older drug user's home] *than some lavatory somewhere"*. Furthermore, the interviewee alluded to the fact that his younger friend might benefit financially be referring to his own social security payments in relation to *"the money we get"*. These findings show a high level of adaptation and resilience among older drug users in relation to social support and we recommend further research should explore these relationships in more detail.

In general, ageing is associated with a decline in memory [34] and interviewees were specifically asked about this. Problems with memory, confirmed by six interviewees, were particularly related to what they termed 'short term' memory, for example, putting an object down and not remembering where it was placed a few minutes later. Of those who commented, two felt that age had contributed to their lack of memory, while most felt this was more associated with drug and alcohol use; *"I can only put it down to just, down to drug abuse and drink abuse, really. I can't really put it down to anything else"*. The older interviewee detailed in the paragraph above had a significantly impaired memory and relied greatly upon his younger male friend/carer. Of particular importance, the interviewee relied upon this friend to remember to collect his methadone prescription, a drug which the older interviewee needed to consume daily. We know that high levels of alcohol consumption result in memory impairment due to thiamine deficiency, and the risk of even mild cognitive impairment in later life is significantly elevated among those who consume alcohol frequently compared to those who drink infrequently [35]. Continuing drug use into older age may carry particular risks in terms of the concurrent effect on the brain; the brain changes in a number of ways across the life course, and the effect of these changes when combined with substance use are not well understood [13].

### Health service contact

Interviewees discussed contact with both generic and specialist services. Care received in hospitals was not always optimal and some who had received care within this setting perceived hospital staff to treat them differently because they were drug users; *"Like normally when you go into hospital and they find out that you're a heroin addict, they normally don't want to know you"*. One man found his experience of hospital particularly humiliating with staff assuming he had hidden drugs within the toilet; *"And they come in one night and says they couldn't find the toilet I put it *[the drugs] *in. In front of everybody and they put it in one of them big needles and squirted into my mouth in front of everybody. Makes you feel that big"*. In another instance, a confrontation caused by the misdiagnosis of a broken leg resulted in the police being called;

*"They didn't diagnose it as a broken leg. They said there was nothing on the x-ray and I was in agony, y'know, with a walking stick and a broken leg. And I had to go three times to the hospital y'know... And I went back and they missed it again. And by then they're getting the police to throw me out the hospital"*.

The above negative accounts of experiences were countered by descriptions of instances of being cared for by healthcare staff with compassion and sensitivity; *"the staff on that ward, they were good to me... I was happy in there. I could have stayed in there"*.

The *de facto *national service framework for drugs and alcohol highlights the importance of integrated care pathways and including non-drug specific services such as hospitals in the treatment of drug users; in particular in screening for drug use and referral into specialist services [36]. While this integration is important for drug users of all ages, older drug users are more likely than their younger counterparts to be in contact with hospitals for a number of general chronic conditions, in addition to those specifically associated with their drug use. Hospitals therefore offer additional opportunities for screening and referral for older drug users and it is important that stigmatisation does not deter them from seeking hospital assistance.

Finally, with respect to hospitals, one man complained that doctors would not prescribe his dying friend, also a drug user, analgesia because he was using methadone as part of his drug treatment. The safe use of opioids to manage pain does present a challenge, but there are a number of reasons why drug users may have greater needs than non-drug users regarding pain management, and drug use *per se *should not preclude a practitioner prescribing an opioid. In particular, people with a history of drug use that need palliative care, should not be denied opioids to manage pain, and often these will need to be given in conjunction to drugs received as part of substitution therapy such as methadone [37]. While mentioned by a single interviewee with only indirect experience, pain management and palliative care are likely to become important issues as a growing number of drug users reach older age and stigma and discrimination should not be allowed to affect care.

Other non-drug related care discussed by interviewees included generic rehabilitative treatment and this tended to be perceived as poor. The man who presented at hospital with a broken leg, for example, bemoaned; *"I can't get a physiotherapist to come out. Or she did and she says 'oh that's great progress'. Just to tick me off the list y'know"*. A second man, who found the impaired verbal skills that followed his stroke very frustrating, had been offered, but had not accepted, speech therapy. He appeared to lack the understanding that such therapy could be advantageous; *"I think my stroke will get better on its own if it's going to get better. I really do. I don't think anybody can do anything about it"*. It is a well accepted phenomenon that the least advantaged in society often make the least demands of specialist health services [38] despite their needs and our results confirm this; interviewees tended to have poor health but low expectations of specialist health services.

With respect to the views expressed by the interviewees about their care within drug services, no issues unique to older drug users emerged except for one man who said of his recent experience in residential rehabilitation that he was *"a bit embarrassed, y'know, being the oldest one" *and a second interviewee commented that there were many more services available to drug users now compared to when he had been younger. Interviewees were complimentary about outreach staff (who visit drug users in their homes and usually make few demands of their clients) and non-clinical drug service staff; *"This place *[drug service] *is sound *[great]. *They're all sound in here. Like the girl who I see*, [member of staff's name], *she's brilliant her... nothing bad to say about her"*. However, some complained about the doctors. One man, for example, was distressed and complained that a doctor would not increase his prescription of methadone ampoules, only his methadone 'linctus' which he complained made him vomit. Doctors in the UK are advised to prescribe methadone in an oral formulation and not injectable ampoules [26] because injecting carries a greater risk of dying from acute drug toxicity, and this situation demonstrates the difficulties clinicians face in terms of offering care in accordance with clinical guidelines, while still meeting the needs of their clients.

## Conclusion

Qualitative research makes no claims to generalisability; this study was carried out with white, predominantly male drug users in Merseyside, UK and included only ten people. Furthermore, these drug users were recruited through drug services and were all, or had been, problematic drug users (users of opiates and/or crack cocaine) and may not be representative of drug users not in contact with such services or users of different drugs. However, these findings add to knowledge and understanding of the physical and mental health of older drug users and their perceptions of healthcare. We show that in general, drug users aged over 50 have poor levels of physical and mental health; our findings support the premise that substance use into older age exacerbates, or accelerates the onset of, medical conditions which are more prevalent in older age. Consequently, in relation to their needs, our interviewees appear to fit into the 'frail older people' category which is normally associated with people of a much older age [15].

We also highlight cases where drug users perceive their generic healthcare to be sub-optimal and conclude that older drug users, like other lower socio-economic groups, have relatively poor expectations of specialist healthcare. How drug services adapt to this ageing population of drug users and how geriatric services adapt to an increasing number of older people who use drugs need urgent consideration. People interviewed in the present study were recruited through drug services and those older people who are drug dependent and not in contact with services may be presumed to have even greater unmet needs.

Ageing drug users continue to be a neglected sub-group in the UK, largely because of societal attitudes about the behaviours deemed relevant to older people. The recently published national drug strategy [12] for example, failed to mention this change in demographic nor how services should respond to this challenge. This issue is not unique to the UK; the number of older drug users living in the UK and other developed nations will rise [7] and further work is urgently needed to evaluate what challenges this change in demographic presents.

## Competing interests

The authors declare that they have no competing interests.

## Authors' contributions

CMB and BR conceived of the study. LP recruited the participants and conducted the interviews. All authors contributed to the analysis and writing of the manuscript.

## Pre-publication history

The pre-publication history for this paper can be accessed here:


